# Insights on Regioselective Synthesis of Fused Thiazoles: Density Functional Theory Calculations, Local Reactivity Indices, and Molecular Electrostatic Potential Analysis

**DOI:** 10.1002/open.202500393

**Published:** 2025-08-28

**Authors:** Ghaferah H. Al‐Hazmi, Jehan Y. Al‐Humaidi, Sayed M. Riyadh, Mohamed S. M. Ahmed, Magdi E. A. Zaki, Awatif H. Alruwaili, Khaled A. Alanazi, Rajeh H. Almutairi, Sobhi M. Gomha

**Affiliations:** ^1^ Department of Chemistry College of Science Princess Nourah bint Abdulrahman University P.O.Box 84428 Riyadh 11671 Saudi Arabia; ^2^ Department of Chemistry Faculty of Science Cairo University Cairo 12613 Egypt; ^3^ Department of Chemistry Faculty of Science Imam Mohammad Ibn Saud Islamic University (IMSIU) Riyadh 11623 Saudi Arabia; ^4^ Department of Chemistry College of Science Northern Border University Arar 91431 Saudi Arabia; ^5^ Medical Center Islamic University of Madinah Madinah 42351 Saudi Arabia; ^6^ Department of Chemistry Faculty of Science Islamic University of Madinah Madinah 42351 Saudi Arabia

**Keywords:** dehydrative cyclization, density functional theory‐gauge‐including atomic orbital approach, fused thiazoles, enones, molecular electrostatic potential mapping, molecular docking studies

## Abstract

A regioselective protocol is developed and validated for the synthesis of pyrazolo[3,4‐d]thiazoles and polycyclic‐fused thiazoles through the reactions of 2‐[((E)‐benzylidene)hydrazono]‐5‐[(Z)‐4‐methoxybenzylidene]thiazolidin‐4‐one with hydrazine derivatives or heterocyclic amines, respectively. The products are confirmed by spectral and elemental analyses. Density functional theory studies, including frontier molecular orbital analysis, local reactivity indices, and molecular electrostatic potential mapping, explain the observed regioselectivity and support a proposed reaction mechanism. Molecular docking shows that several derivatives (e.g., **6c, 6d**) have strong binding to *S. aureus*, *E. coli*, and topoisomerase II*α*, with energies comparable to standard drugs. Absorption, distribution, metabolism, excretion, and toxicity predictions indicate good oral bioavailability, low blood–brain barrier permeability, and acceptable safety, suggesting these compounds as promising antibacterial and anticancer candidates.

## Graphical abstract

A regioselective synthesis of fused thiazoles was achieved via aza‐Michael cyclization of thiazolidinone derivatives with hydrazines or heteroaryl amines. Density functional theory, local reactivity indices, and molecular electrostatic potential mapping are used to explain selectivity. Docking and absorption, distribution, metabolism, excretion, and toxicity studies suggest promising antibacterial and anticancer potential of selected derivatives.

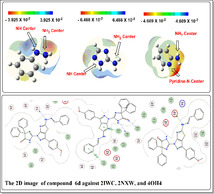

## 1 Introduction

In medicinal chemistry, nitrogen‐ and sulfur‐containing heterocycles, such as thiazoles and pyrazoles, serve as foundational scaffolds for numerous bioactive compounds.^[^ ^1^ ^]^ Thiazoles are five‐membered rings containing one nitrogen and one sulfur atom,^[^ ^2^ ^]^ whereas pyrazoles feature two adjacent nitrogen atoms within a five‐membered ring framework.^[^ ^3^ ^,^ ^4^ ^]^ These heterocyclic motifs are prevalent in natural products and pharmaceutical agents. Molecular hybridization—combining distinct pharmacophores into a single entity—has emerged as a powerful strategy for drug discovery, with thiazole–pyrazole hybrids gaining prominence as versatile platforms for developing novel therapeutics.^[^ ^5^ ^,^ ^6^ ^]^

Thiazole derivatives exhibit diverse biological activities, including antimicrobial, anti‐inflammatory, central nervous system‐modulating, antineoplastic, and anticancer effects.^[^ ^7^, ^8^, ^9^, ^10^, ^11^, ^12^, ^13^, ^14^, ^15^, ^16^, ^17^ ^]^ Pyrazole derivatives similarly demonstrate antimicrobial, anti‐inflammatory, anticancer, antiviral, antidepressant, and anti‐HIV properties.^[^ ^18^, ^19^, ^20^, ^21^, ^22^, ^23^ ^]^ Hybrid molecules integrating both thiazole and pyrazole rings have shown enhanced efficacy, particularly as antimicrobial^[^ ^24^, ^25^, ^26^ ^]^ and anticancer agents.^[^ ^27^, ^28^, ^29^, ^30^ ^]^

**Figure 1:**
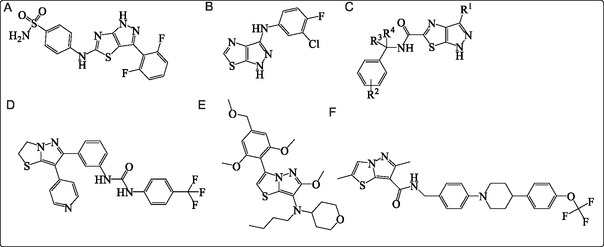
Pharmacologically active pyrazolo‐thiazole derivatives (A–F).

Pyrazolothiazoles have emerged as privileged scaffolds in heterocyclic and medicinal chemistry owing to their broad spectrum of biological activities, including antimicrobial, anticancer, anti‐inflammatory, anti‐HIV, and enzyme inhibitory effects.^[^ ^31^, ^32^, ^33^, ^34^, ^35^, ^36^, ^37^, ^38^ ^]^ Their fused‐ring system imparts conformational rigidity and promotes favorable *π*–stacking interactions, both of which are critical for efficient target binding. Several biologically active pyrazolothiazole derivatives have been reported, demonstrating diverse pharmacological properties. As illustrated in **Figure** **1**, compounds A–C are pyrazolo[4,3‐*d*]thiazole derivatives, where A functions as a cyclin‐dependent kinase inhibitor,^[^ ^39^ ^]^ B as an mGluR4 receptor modulator,^[^ ^40^ ^]^ and C as a generic protein kinase inhibitor scaffold.^[^ ^41^ ^]^ Compounds D–F are pyrazolo[3,4‐*d*]thiazole derivatives, with D serving as a protein kinase inhibitor for cancer therapy,^[^ ^42^ ^]^ E acting as a potent corticotropin‐releasing factor 1 (CRF_1_) receptor antagonist,^[^ ^42^ ^]^ and F exhibiting strong suppressant activity against the *Mycobacterium tuberculosis* H37Ra strain.^[^ ^42^ ^]^ Collectively, these examples highlight the structural diversity and significant therapeutic relevance of pyrazolothiazole frameworks across oncology, neuropharmacology, and infectious disease treatment (Figure 1).

Xanthine oxidase (XO) enzyme in mammals is present in brain, plasma, kidney, liver, and intestine,^[^ ^43^ ^]^ where it is responsible for the conversion of hypoxanthine into xanthine and further conversion of xanthine into uric acid.^[^ ^44^ ^]^ An increasing level of XO leads to aging, renal stone formation, inflammation, carcinogenesis, and hepatitis.^[^ ^45^ ^]^ Overproduction of uric acid leads to gout disease.^[^ ^46^ ^]^ Xanthine oxidoreductase (XOR) can irreversibly coordinate with xanthine and block the terminal step in uric acid biosynthesis.^[^ ^47^ ^]^ Pyrazolothiazolopyrimidine, as a tri‐heterocyclic fused system, was found to have inhibitory activity against xanthine oxidase similar to the drug allopurinol.^[^ ^48^ ^]^ Additionally, these scaffolds have been investigated in the field of infectious diseases as antimicrobial^[^ ^49^ ^,^ ^50^ ^]^ agents. The most effective synthetic strategies for assembling pyrazolothiazolopyrimidine involve multi‐component reactions in the presence^[^ ^51^ ^,^ ^52^ ^]^ or absence^[^ ^53^ ^]^ of solvents, including the formation of C—N and C—C bonds for molecular complexity.

**Figure 2:**
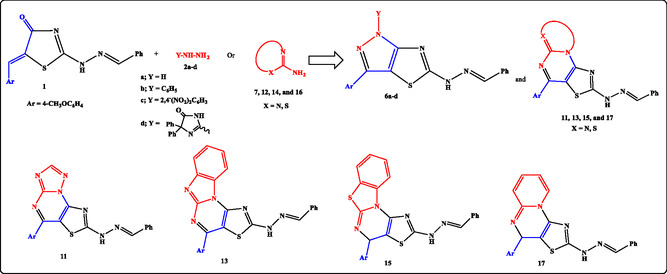
Targeted pyrazolo‐thiazole derivatives.

Prompted by the privileged pharmacological activities of pyrazolothiazoles and pyrazolothiazolopyrimidines, and in continuation of our research aimed at the synthesis of bioactive heterocycles,^[^ ^54^, ^55^, ^56^, ^57^, ^58^ ^]^ we designed and synthesized a series of target molecules in which the thiazolone ring, bearing two electrophilic sites (CO–CH=CH), undergoes regioselective cyclization with nucleophilic centers (NH_2_, NH) of hydrazines or heterocyclic amines, followed by dehydration. The resulting fused pyrazolo[3,4‐d]thiazoles and polycyclic analogs, as illustrated in **Figure** **2**, were subsequently characterized and evaluated. Density functional theory (DFT) calculations and molecular docking studies were employed to rationalize the observed regioselectivity, structural features, and predicted binding interactions.

## 2 Results and Discussion

**Scheme 1:**
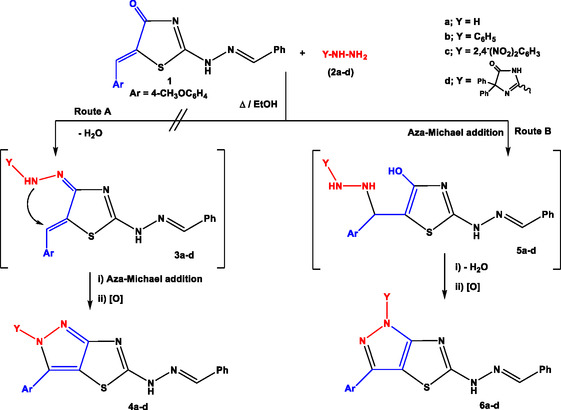
Synthesis of pyrazolo[3,4‐*d*]thiazoles.

The study was inaugurated by treatment of 2‐[((E)‐benzylidene)hydrazono]‐5‐[(Z)‐4‐methoxybenzylidene]thiazolidin‐4‐one (**1**)^[^ ^59^ ^]^ with hydrazine hydrate (**2a**) or hydrazine derivatives (**2b–d**) in absolute ethanol under refluxing conditions **Scheme** **1**). The reaction proceeded via two possible strategies.

The first pathway (Route A) involves the formation of hydrazone intermediate (**3a‐d**) which undergoes synchronous aza‐Michael addition and oxidation to give the respective regio‐isomer 2*H*‐pyrazolo[3,4‐*d*]thiazole (**4a‐d**). The other pathway (Route B) involves the nucleophilic addition of (NH_2_) group into exocyclic (C=C) leading to formation of Michael adduct (intermediate **5a‐d**) followed by dehydration and subsequent oxidation to give the other regio‐isomer 1*H*‐pyrazolo[3,4‐*d*] thiazole (**6a‐d**), as depicted in Scheme 1.

**Scheme 2:**
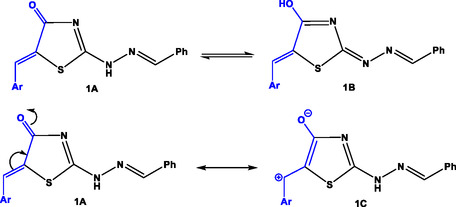
Tautomeric and resonating forms of compound **1.**

Thiazolidin‐4‐one (**1**) could be formulated in two tautomeric forms (Keto‐form **1A**, and enol‐form **1B**) and two resonance forms (**1A** and **1C**), as shown in **Scheme** **2**. Thus, the carbonyl group in thiazolidinone ring has weak electrophilic character due to its conjugation with exocyclic (C=C) and enolization with hydrazone moiety. As a result of that reason, condensation step in Route A could not be performed at the initial step, and consequently, Route A was ruled out. In Route B, aza‐Michael 1,4‐addition was most probably occurred followed by condensation of (NH) with enolic (OH) group to give the isolated regio‐isomer **6a‐d** as reported in the literature.^[^ ^60^ ^]^

**Scheme 3:**
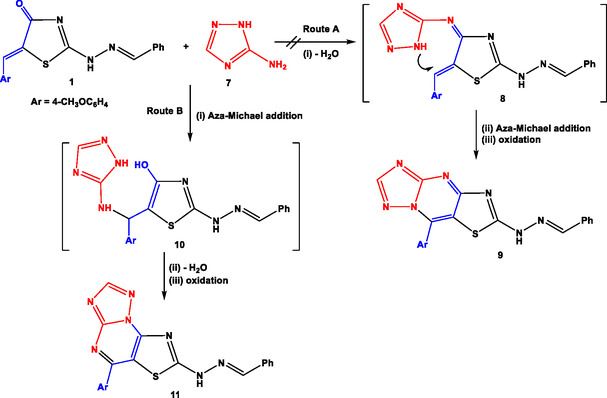
Synthesis of thiazolo‐triazolo‐pyrimidine.

Similarly, 2‐[((*E*)‐benzylidene)hydrazono]‐5‐[(*Z*)‐4‐methoxybenzylidene]thiazolidin‐4‐one (**1**) was reacted with 5‐amino [1,2,4]triazole (**7**) under the same conditions to furnish thiazolo[4,5‐*d* [1,2,4] triazolo[1,5‐*a*]pyrimidine (**9**) or thiazolo[5,4‐*e* [1,2,4] triazolo[1,5‐*a*]pyrimidine (**11**), as shown in **Scheme** **3**.

Basically, the nucleophilicity of amino group at position 5 in triazole ring is more than that of an endocyclic (NH) group due to the participation of lone pair of electrons on (NH) in the aromatization of triazole ring.^[^ ^61^ ^,^ ^62^ ^]^ Thus, the amino group (NH_2_) initiated the reaction mechanism (Scheme 3) and underwent aza‐Michael 1,4‐addition onto the electron‐deficient (C=C) (Route B) to give the intermediate **10**. Dehydration of the endocyclic (NH) group with enolic (OH) followed by oxidation of the latter intermediate afforded compound **11** as the isolated product. Route A was excluded due to the lower electrophilicity of carbonyl group as reported in literature.^[^ ^63^ ^,^ ^64^ ^]^ The concluding evidence for the structural elucidation of regio‐isomer **11** was earned from spectroscopic data. Its ^1^H‐nuclear magnetic resonance (NMR) spectrum revealed four singlet signals at *δ*=3.86, 8.37 (2H), and 11.95 ppm attributed to (OCH_3_), (CH=N), CH‐triazolo[1,5‐*a*] pyrimidine,^[^ ^65^ ^]^ and (NH) protons, respectively.

**Scheme 4:**
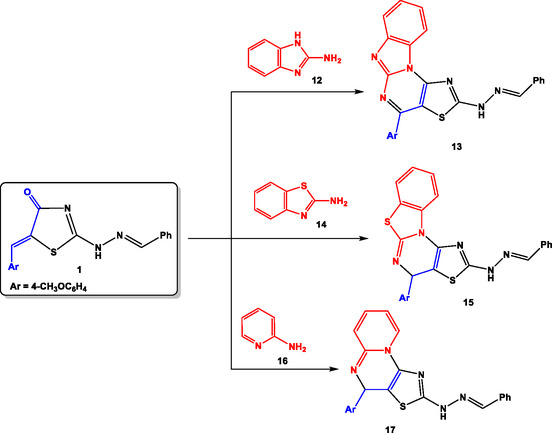
Synthesis of polycyclic fused thiazole.

Utilizing the experimental conditions, we have generalized the substrate scope in our methodology by the reaction of compound **1** with 2‐aminobenzimidazole (**12**), 2‐aminobenzothiazole (**14**), and 2‐aminopyridine (**16**) via dehydrative cyclization to give the respective products benzoimidazo[1,2‐*a*]thiazolo[5,4‐*e*]pyrimidine (**13**), benzothiazolo[3,2‐*a*]thiazolo[5,4‐*e*]pyrimidine (**15**), and pyrido[1,2‐*a*]thiazolo[5,4‐*e*]pyrimidine (**17**) (**Scheme** **4**). All the synthesized products were characterized by elemental analyses and spectral date (infrared (IR), NMR, and mass spectrometry (MS)).

## 3 Computational Investigations

### 3.1 Molecular Electrostatic Potential (MEP)

All quantum chemical calculations were performed using *Gaussian 09, Revision D.01*. Geometry optimizations and electronic property calculations were conducted at the B3LYP/6‐31+G(d, p) level of theory. The RB3LYP functional was used for closed‐shell systems, while UB3LYP was employed for open‐shell species. MEP surfaces were generated using an isovalue of 0.0004 and a fine grid with a step size of 0.2 Bohr, ensuring accurate charge distribution visualization.

MEP maps were calculated to situate the most electrophilic site in thiazolidine‐4‐one (**1**) and the most nucleophilic site in hydrazines (**2b‐d**) or heterocyclic amines (**7, 12, 14**, and **16**). Thus, the regioselectivity of their reactions could be established. The Gaussian 09 software and the 6–31+G (d, p) basis set were used to evaluate the atomic spin density under unrestricted formalization (UB3LYP) and compute the Parr function.^[^ ^66^ ^,^ ^67^ ^]^ Parr electrophilicity (*P*_*r*_^*+*^), electrophilicity (*ω*), and local electrophilicity (*ωP*_*r*_^*+*^) of the olefinic and carbonyl groups in thiazolidine‐4‐one (**1**) were illustrated in **Table** **1**.

**Table 1:** Local electrophilicity indexes of thiazolidin‐4‐one (1), identifying the most reactive electrophilic site.

Compd.	Site	*P*_*r*_^*+*^	*ω*	*ωP*_*r*_^*+*^
Thiazolidine‐4‐one (1)	CH=CH‐CO	0.029	3.99	0.116
	CO‐CH = CH	−0.027	3.99	−0.108

As depicted in Table 1, the parr and local electrophilicity of olefinic group (0.029 and 0.116) are higher than the carbonyl group (−0.027 and 0.108) in thiazolidine‐4‐one (**1**) which indicates the most electron‐deficient center in enone system.

In a similar manner, parr nucleophilicity (*P*_*r*_^−^), nucleophilicity (N), and local nucleophilicity (*NP*_*r*_^−^) of hydrazines (Ar‐NHNH_2_) and heterocyclic amines were measured and tabulated in **Table** **2**.

**Table 2:** Local nucleophilicity indexes of hydrazines and heterocyclic amines, showing the most reactive nucleophilic centers.

Compd.	Site	*P*_*r*_^−^	N	*NP*_*r*_^−^
Phenyl hydrazine (2b)	NH_2_‐	0.001	4.16	0.004
	NH‐	−0.002	4.16	−0.008
2,4‐Dinitrophenyl hydrazine (2c)	NH_2_‐	−0.0001	2.76	−0.0003
	NH‐	−0.016	2.76	−0.0442
2‐Hydrazinyl‐5,5‐diphenyl‐3,5‐dihydro‐4*H*‐imidazol‐4‐one (2d)	NH_2_‐	−0.0118	3.24	−0.0382
	NH‐	−0.2053	3.24	−0.6652
5‐Amino [1,2,4]triazole (7)	NH_2_‐	0.277	3.22	0.892
	NH (endocyclic)	0.121	3.22	0.389
2‐Aminobenzimidazole (12)	NH_2_‐	0.002	3.97	0.008
	NH (endocyclic)	−0.005	3.97	0.020
2‐Aminobenzothiazole (14)	NH_2_‐	0.031	3.67	0.114
	N (endocyclic)	−0.058	3.67	−0.213
2‐Aminopyridine (16)	NH_2_‐	0.265	3.68	0.975
	N (endocyclic)	−0.002	3.68	−0.007

The data in Table 2 deduced that the nucleophilicity of (NH_2_) group in hydrazines and heterocyclic amines is more than (NH) group and endocyclic (NH or N), respectively. Thus, the reactions will be initiated when the olefinic site (C=C) in compound **1** is regio‐selectively attached by (NH_2_) group (Route B in Scheme 1 and 2).

**Figure 3:**
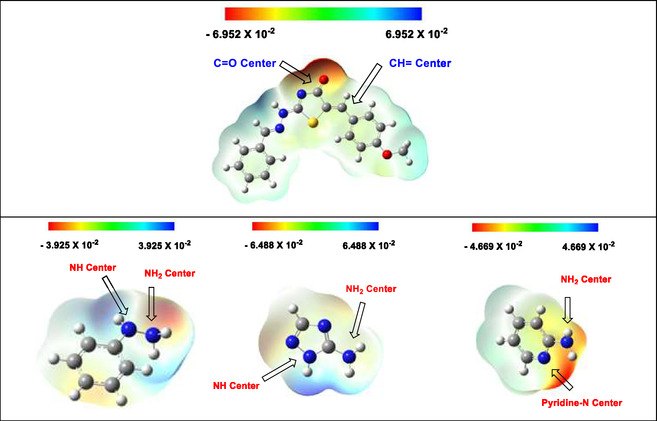
MEP mapping of compounds **1**, **2b**, **7**, and **16** (B3LYP/6.31G(d,p); isoval = 0.0004.

In MEP maps, the potential decreased in the following order: red, orange, yellow, green, and blue attributed to the electron density of each atom in the molecule.^[^ ^61^ ^]^ The blue region promoted the electrophilic sites, the red and yellow regions promoted the nucleophilic sites while the green region promoted the neutral sites with zero potentials.^[^ ^68^ ^]^ **Figure** **3** illustrates the MEP maps of thiazolidine‐4‐one (**1**), phenyl hydrazine (**2b**), 5‐amino [1,2,4]triazole (**7**), and 2‐aminopyridine (**16**) as representative examples to comprehend the locations of electrophilic and nucleophilic centers.

### 3.2 Global Descriptors

The total electronic energy E (RB3LYP), as a thermodynamic parameter, could be used to manifest the energies of different regio‐isomers from Route‐A (**4a‐4c, 9**) or Route‐B (**6a‐6c, 11**) was calculated. **Table** **3** also presents the global reactivity descriptors for these regio‐isomers, including ionization energy (I), electrophilicity (*ω*), electron affinity (A), global softness (S), global hardness (*η*), chemical potential (μ), and nucleophilicity (N).

**Table 3:** Global reactivity descriptors and total electronic energies of regio‐isomers from Routes A and B.

No.	Highest occupied molecular orbital (HOMO)	Lowest unoccupied molecular orbital (LUMO)	I = −E_HOMO_	A = −E_LUMO_	η = [E_L_ − E_H_]/2	S = 1/*η*	*µ* = −(I + A)/2	*ω* = *µ* ^2^/2*η*	N = E_HOMO_−E_HOMO_TCE	E (RB3LYP)[Kcal mol^−1^]
4a	−5.37	−0.76	5.37	0.76	2.31	0.43	3.07	2.04	4.04	−846,292.76
6a	−5.27	−0.78	5.27	0.78	2.25	0.45	3.03	2.04	4.14	−846,294.11
4b	−5.29	−1.49	5.29	1.49	1.90	0.53	3.39	3.02	4.12	−1,049,910.04
6b	−5.18	−1.68	5.18	1.68	1.75	0.57	3.43	3.36	4.23	−1,049,915.96
4c	−5.76	−2.86	5.76	2.86	1.45	0.69	4.31	6.41	3.65	−1,306,551.01
6c	−5.71	−2.77	5.71	2.77	1.47	0.68	4.24	6.11	3.70	−1,306,556.46
9	−5.90	−1.93	5.90	1.93	1.99	0.50	3.92	3.86	3.51	−1,021,466.11
11	−5.80	−1.98	5.80	1.98	1.91	0.52	3.89	3.96	3.61	−1,021,466.66

It is known that, the more negative value of the total electronic energy reflected more stability of the investigated molecule. As shown in Table 3, regio‐isomers **6a** (−846,294.11 Kcal/mol), **6b** (−1,049,915.96 Kcal/mol), **6c** (−1,306,556.46 Kcal/mol), and **11** (−1,021,466.66 Kcal/mol) are more stable than regio‐isomers **4a** (−846,292.76 Kcal/mol), **4b** (−1,049,910.04 Kcal/mol), **4c** (−1,306,551.01 Kcal/mol), and **9** (−1,021,466.11 Kcal/mol), respectively, which established our proposed mechanism in Schemes 1–3.

### 3.3 Molecular Docking Investigation

For molecular docking, *S. aureus* (PDB: 2IWC) and *E. coli* (PDB: 2NXW) were selected as representative gram‐positive and gram‐negative bacterial targets due to their clinical relevance and high‐resolution crystal structures. Topoisomerase II*α* (PDB: 4OH4) was chosen as a validated anticancer target, as its inhibition disrupts DNA replication in rapidly dividing cells. These proteins were selected to evaluate both antibacterial and anticancer potential and to identify the dominant mode of action. Docking results revealed that several compounds (particularly **6c** and **6d)** interacted favorably with all targets; however, stronger binding affinities and more consistent interactions with 4OH4 suggest that anticancer activity is the primary mode of action, with selective compounds also exhibiting promising antibacterial potential.

The Molecular Operating Environment (MOE) software version 2022.02 was utilized for the molecular docking analysis. The interaction between the four proteins (PDB: 2IWC, 2NXW, and 4OH4) is used to assess the biological activity of the investigated compounds (**6a‐d, 11, 13, 15,** and **17**) using gentamicin and ampicillin as reference antibiotics. The most crucial factors in comparing the ligand compounds are the binding energy, the binding score, and the number of hydrogen bond interactions.

### 3.4 Antibacterial Activity

**Figure 4:**
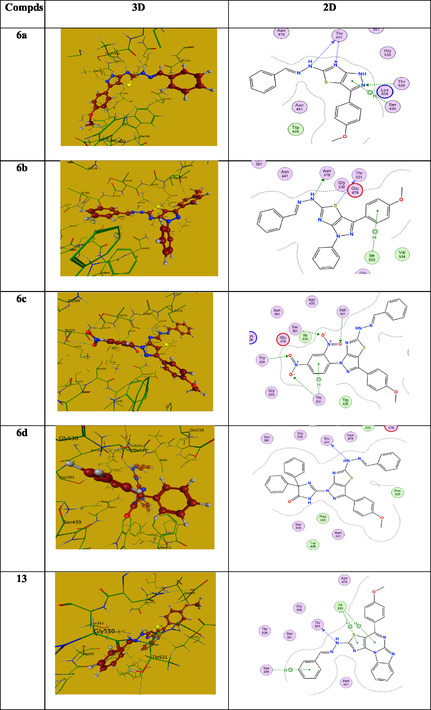
The 2D and 3D images of compounds **6a‐d** and **13** against 2IWC.

Initially, we studied the gram‐positive bacteria‐protein (2IWC)‐ ampicillin interaction. It revealed binding energy (S = −6.073 Kcal/mol) with root mean square deviation (RMSD) = 1.39 Å. Ampicillin's interaction with 2IWC revealed three hydrogen bonds (two H‐acceptors and one H‐donor) with the side chain amino acids THR531 and ILE533, two hydrogen bonds (H‐acceptors) with the main chain amino acids SER439 and ASN441, and one hydropic bond (pi‐H) with ASN478 (**Table** **4**). Compound **6a** as a model example revealed binding energy (−5.72 Kcal/mol) and RMSD = 1.8 Å. It displayed three H‐bonds (one H‐acceptor one H‐donor) with side chain amino acid THR531 and one H‐acceptor with main chain amino acid THR529 in addition to one hydrophobic bond (pi‐H) with main chain amino acid SER439. Table 4 summarizes the data collected clarifying the protein‐ligand interactions of the most active candidates. The observations showed that all compounds under investigation are joined with the same amino acid(s) as ampicillin, which supports the biological efficacy of the promising candidates against 2IWC protein. **Figure** **4** illustrates the 2D and 3D images of the tested compounds.

**Table 4:** Docking interactions and binding energies of 6a–d, 13, and ampicillin with *S. aureus* (2IWC).

	Ligand–protein interactions/type of interactions	Binding energy [Kcal mol^−1^]	Number of H‐bond	RMSD [Å]
6a	THR531 (A)/H‐donorTHR531 (A)/H‐acceptorTHR529 (A)/H‐acceptorSER439 (A)/pi‐H	−5.72	3	1.8
6b	THR531 (A)/H‐donorASN478 (A)/H‐donorILE533 (A)/pi‐H	−6.19	3	2.1
6c	THR529 (A)/H‐acceptorTHR531 (A)/H‐acceptorASN441 (A)/H‐acceptorSER391 (A)/H‐acceptorTHR531 (A)/pi‐H	−7.23	4	2.5
6d	THR531 (A)/H‐donor	−7.18	1	1.9
13	THR531 (A)/H‐donorSER439 (A)/pi‐HILE533 (A)/pi‐HILE533 (A)/pi‐H	−5.91	1	1.3
Ampicillin	THR531 (A)/H‐donorTHR531 (A)/H‐acceptorASN441 (A)/H‐acceptorILE533 (A)/ H‐acceptorSER439 (A)/H‐acceptorASN478 (A)/pi‐H	−6.07	5	1.39

**Figure 5:**
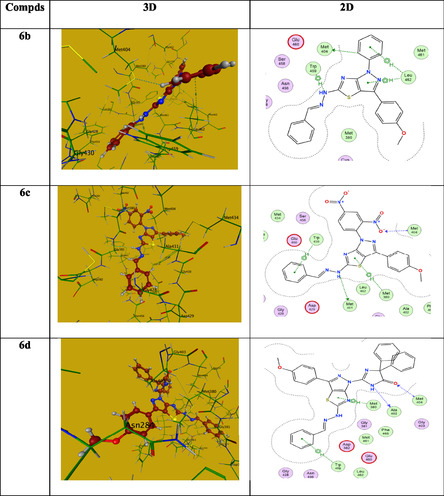
The 2D and 3D images of **6b, 6c,** and **6d** against 2NXW.

Similarly, we evaluated the ligand–protein interaction of the candidates under investigation with 2NXW as a gram‐negative bacteria protein. The collected data are summarized in **Table** **5**, and the 2D and 3D images are gathered in **Figure** **5**. The interaction of gentamicin with the 2NXW protein was investigated. Four hydrogen bonds were observed through the amino acids MET404, MET434, and ALA402, with binding energy of (−5.17 Kcal/mol) and RMSD = 1.5 Å. Compound **6d**, as a representative example, interacts with 2NXW with a binding energy of (−7.63 Kcal/mol) and an RMSD of 2.0 Å. Its interaction with 2NXW revealed two H‐bonds (one H‐acceptor) with the side chain amino acid MET404 and (one H‐donor) with side chain amino acid ALA402, and two hydrophobic bonds (pi‐H) with main chain amino acids MET380 and TRP459. The investigated compounds are linked to the same amino acid(s) as the standard candidate (gentamicin), supporting the tested compounds’ potential biological efficacy towards gram‐negative bacteria (Table 5 and Figure 5).

**Table 5:** Docking interactions and binding energies of 6b–d and gentamicin with *E. coli* (2NXW).

	Ligand–protein interactions/type of interactions	Binding energy [Kcal mol^−1^]	Number of H‐bond	RMSD [Å]
6b	MET404 (A)/H‐donorTRP459 (A)/H‐piLEU462 (A)/pi‐HLEU462 (A)/pi‐H	−7.04	1	1.5
6c	MET461 (A)/H‐donorMET404 (A)/H‐acceptorMET380 (A)/pi‐HTRP459 (A)/pi‐H	−7.24	2	0.9
6d	ALA402 (A)/H‐donorMET404 (A)/H‐acceptorMET380 (A)/pi‐HTRP459 (A)/pi‐H	−7.63	2	2.1
Gentamicin	MET434 (A)/H‐donorMET404 (A)/H‐donorMET404 (A)/H‐donorALA402 (A)/H‐donor	−5.17	4	1.5

For the antibacterial targets, structure–activity relationships (SAR) analysis indicated that compounds bearing electron‐donating methoxy groups, such as **6a**, maintained moderate binding through hydrogen bonding with conserved active‐site residues, while electron‐withdrawing substituents, as in **6c**, enhanced binding affinity against *S. aureus* (2IWC) via additional polar contacts. Bulky heteroaryl substitutions, exemplified by **6d**, favored hydrophobic interactions within *E. coli* (2NXW) binding pockets, correlating with improved predicted antibacterial activity.

### 3.5 Antitumor Activity

**Figure 6:**
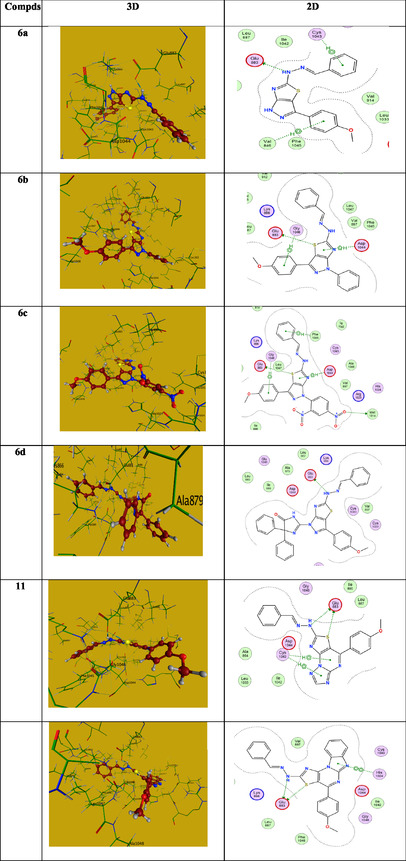
The 2D and 3D images of **6a‐d, 11,** and **13** against 4OH4.

The interaction between the protein (PDB: 4OH4) and the investigated compounds is utilized to assess the antitumor activity using doxorubicin as a benchmark antibiotic. The binding affinities were determined by analyzing hydrogen bond formation, binding energy, and the spatial orientation of the ligand relative to key amino acid residues within the protein. As retrieved in **Table** **6**, the interaction between the widely used antibiotic Doxorubicin and antitumor protein (4OH4) revealed binding energy (−7.21 Kcal/mol) with RMSD = 1.87 A^o^ via three hydrogen bonds (two H‐donors) with the main chain amino acid GLU883 with a distance apart 2.93 and 2.79 Å and one H‐acceptor with CYS917 (2.98 Å distance), and one pi‐H hydrophobic bond with LEU838 with a distance apart 3.83 Å. Compound **13** as a representative example, revealed binding energy (−7.80 Kcal/mol) with RMSD = 2.0 Å. It displayed two H‐bond donors with main chain amino acids GLU883 and one hydrophobic bond with HIS1024. The data revealed that all the investigated compounds are linked with the same amino acid (GLU883), such as the reference antibiotic doxorubicin, with similar binding energies (−6.52 to—8.41 Kcal/mol) (Table 6 and **Figure** **6**).

**Table 6:** Docking interactions and binding energies of 6a–d, 11, 13, and doxorubicin with topoisomerase II*α* (4OH4).

	Ligand–protein interactions/type of interactions	Binding energy [Kcal mol^−1^]	Number of H‐bond	RMSD [Å]
6a	GLU883 (A)/H‐donorVAL846 (A)/pi‐HCYS1043 (A)/pi‐H	−7.73	1	1.6
6b	GLU883 (A)/H‐donorASP1044 (A)/pi‐HGLY1046 (A)/pi‐H	−7.94	1	2.4
6c	GLU883 (A)/H‐donorMET1014 (A)/H‐donorASP1044 (A)/pi‐HPHE1045 (A)/pi‐HGLY1046 (A)/pi‐H	−7.52	2	2.1
6d	GLU883 (A)/H‐donor	−8.41	1	1.1
11	GLU883 (A)/H‐donorGLU883 (A)/H‐donorCYS1043 (A)/pi‐HCYS1043 (A)/pi‐H	−6.52	2	1.8
13	GLU883 (A)/H‐donorGLU883 (A)/H‐donorHIS1024 (A)/pi–pi	−7.80	2	2.0
Doxorubicin	GLU883 (A)/H‐donorGLU883 (A)/H‐donorCYS917 (A)/H‐acceptorLEU838 (A)/pi‐H	−7.21	3	1.8

The docking results revealed clear SAR, where substitutions on the pyrazolo[3,4‐d]thiazole core influenced binding affinity and interaction profiles. Electron‐withdrawing groups, such as nitro moieties in compound **6c**, enhanced hydrogen bonding and *π*–*π* interactions with active‐site residues, leading to higher binding energies. Bulky aromatic substitutions, as in **6d**, improved hydrophobic contacts, particularly within the topoisomerase II*α* pocket, correlating with strong predicted anticancer activity. These findings indicate that both electronic effects and steric factors play a key role in modulating biological activity.

Comparative analysis of the docking results revealed that several compounds exhibited stronger predicted binding affinities toward topoisomerase II*α* (4OH4) than toward the bacterial targets, indicating anticancer activity as the dominant mode of action. Nonetheless, compounds such as **6c** and **6d** also showed notable antibacterial potential, suggesting a dual activity profile for selected derivatives.

## 4 In Silico ADME (Absorption, Distribution, Metabolism, and Excretion) and Toxicity Predictions

### 4.1 Drug Likeness Prediction

**Figure 7:**
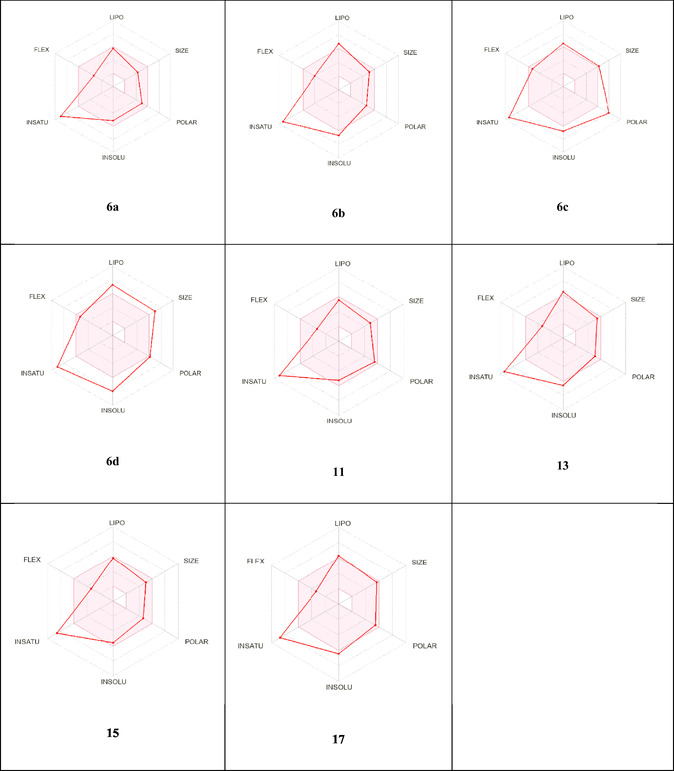
Radar related to physicochemical properties of molecules **6a‐d, 11, 13, 15,** and **17.**

The drug‐likeness tool is a useful method for the preliminary biological evaluation of synthetic drugs. **Table** **7** illustrates the extent to which the synthesized candidates adhere to the Lipinski rule. The data obtained indicates that compounds **6a‐d, 11, 13, 15,** and **17** have perfect alignment of the compounds under investigation with the Lipinski criterion, with no violations with Lipinski, Ghose, Veber, and Egan (See Table 7 and **Figure** **7**).^[^ ^69^ ^]^

**Table 7:** Predicted drug‐likeness properties of synthesized compounds versus standard drugs.

Compound	MWt < 500a)	HBD < 5b)	HBA < 10c)	MlogP < 5d)	RBse)	Lipinski violations	Ghose violations	Veber violations	Egan violations	Muegge violations
6a	349.41	2	4	2.73	5	0	0	0	0	0
6b	425.51	1	4	4.11	6	0	0	0	0	1
6c	515.5	1	8	2.44	8	2	2	1	1	2
6d	583.66	2	6	3.99	8	1	2	0	1	1
11	401.44	1	6	2.77	5	0	0	0	0	0
13	450.52	1	5	3.92	5	0	1	0	0	1
15	413.49	1	4	3.03	5	0	0	0	0	0
17	469.58	1	4	4.05	5	0	1	0	0	1
Levofloxacin	361.37	1	6	2.25	2	0	0	0	0	0
Idarubicin	497.49	5	10	−1.05	3	0	1	1	1	1

a) Molecular weight,

b) number of H‐bond donor,

c) number of H‐bond acceptor,

d) MLogP,

e) number of rotatable bonds.

Further examination of the synthesized compounds revealed that most candidates (**6a‐d, 11, 13, 15,** and **17** derivatives) exhibited topological polar‐surface areas (TPSAs) between 92.57 and 134.03 Å, which fit the acceptable limit (less than 140).^[^ ^70^ ^]^ Furthermore, all tested compounds under investigation reveal remarkable oral bioavailability, with a %absorption (%ABS = 109 −(0.345 × TPSA) close to that of levofloxacin (See **Table** **8**).

**Table 8:** TPSA, % absorption, intestinal absorption, and clearance of synthesized compounds versus standards.

Compound	TPSA < 140	%ABS109 ‐ (0.345 × TPSA)	Intestinal absorption (human)	Total clearance
6a	103.43	73.32	90.33	0.27
6b	92.57	77.06	83.45	0.28
6c	184.21	45.45	100	0.37
6d	134.03	62.76	100	0.46
11	117.83	68.35	95.41	0.31
13	104.94	72.80	85.44	0.34
15	92.04	77.25	89.48	0.27
17	115.65	69.10	94.30	0.14
Levofloxacin	75.01	82.75	94.38	0.23
Idarubicin	176.61	48.06	71.44	0.82

**Table** **9** presents the findings of pharmacokinetic evaluation and medicinal investigation. It can be inferred that all candidates **6a‐d, 11, 13, 15,** and **17** have acceptable bioavailability scores and 0% blood–brain barrier (BBB) permeability.^[^ ^71^ ^]^ Consequently, there are no pan‐assay interference compounds (PAINS) warnings for the three series **6a‐d, 11, 13, 15,** and **17**. The compounds **6a‐d, 11, 13, 15,** and **17** that were studied had synthetic accessibility scores that vary from 3.33 to 5.04, which is close to the 3.63 and 5.63 value for levofloxacin and idarubicin, respectively. This supports that the candidates under evaluation may be accessed for mass production.^[^ ^72^ ^]^

**Table 9:** Pharmacokinetic properties and synthetic accessibility of synthesized compounds versus standards.

Compound	GI absorption	BBB permeant	Pgp substrate	Bioavailability score	PAINS alerts	Synthetic accessibility
6a	High	No	No	0.55	0	3.33
6b	High	No	No	0.55	0	3.64
6c	Low	No	No	0.17	0	4.01
6d	Low	No	No	0.55	0	4.62
11	High	No	No	0.55	0	3.45
13	High	No	No	0.55	0	3.57
15	High	No	No	0.55	0	4.49
17	High	No	No	0.55	0	5.04
Levofloxacin	High	No	Yes	0.55	0	3.63
Idarubicin	Low	No	Yes	0.55	1	5.63

### 4.2 Toxicity Profiles of Synthesized Compounds

The data collected is listed in **Table** **10**. The pkCSM online application was utilized to estimate the toxicity of the synthesized compounds. Likewise, to levofloxacin, none of the compounds under investigation were harmful to AMES (Ames Test (bacterial reverse mutation assay)). In addition, the Oral Rat Acute Toxicity (LD50) values of all the candidates under examination vary from 2.27 to 3.69, which is comparable to the 2.56 and 3.20 values of levofloxacin and idarubicin, respectively. Moreover, the oral rat acute toxicity levels (lowest observed adverse effect levels (LOAELs)) of most investigated compounds ranged from 0.85 to 2.14 which are close to that of levofloxacin and idarubicin (1.38 and 2.48).^[^ ^73^ ^,^ ^74^ ^,^ ^75^ ^]^

**Table 10:** Predicted toxicity profiles of synthesized compounds versus standards.

Compound	AMES toxicity	Max tolerated dose (human)a)	hERG I (human ether‐à‐go‐go‐related gene (potassium ion channel))	hERG II	LD50b)	LOAELc)	Hepatotoxicity	Skin sensitization	T‐Pyriformis toxicityd)	Minnow toxicitye)
6a	No	0.62	No	Yes	2.81	2.14	Yes	No	0.30	1.60
6b	No	0.80	No	Yes	2.39	0.34	Yes	No	0.28	−3.28
6c	Yes	0.24	No	Yes	2.91	−0.29	Yes	No	0.28	−4.81
6d	Yes	0.45	No	Yes	3.69	1.74	Yes	No	0.28	−3.38
11	No	0.08	No	Yes	2.65	0.87	Yes	No	0.23	1.01
13	Yes	0.34	Yes	Yes	2.43	−0.01	No	No	0.23	−0.99
15	No	0.58	No	No	2.28	0.85	No	No	0.83	−2.79
17	No	0.83	No	No	2.27	0.98	Yes	No	0.51	−2.82
Levofloxacin	No	−0.33	No	No	2.56	1.38	Yes	No	0.30	1.65
Idarubicin	Yes	−0.35	No	Yes	3.20	2.48	Yes	No	0.23	3.51

a) log mg/kg/day,

b) mol/kg,

c) log mg/kg_bw/day,

d) log ug/L,

e) log mM.

## 5 Experimental Section

### 5.1 Instruments

Melting points were determined using an Electrothermal Gallenkamp apparatus. Analytical thin‐layer chromatography (TLC) was carried out using Merck silica gel GF254 plates. IR spectra were obtained with a Pye‐Unicam SP300 spectrometer, and elemental analysis was performed on a PerkinElmer 2400 Series II instrument. NMR spectra (¹H at 500 MHz and ¹³C at 125 MHz) were recorded on a JEOL JNM‐ECZR 500 MHz spectrometer, while mass spectrometric analysis was conducted using a Thermo Scientific GC/MS ISQ system.

### 5.2 General Procedure for the Reactions of Thiazolidin‐4‐one (1) with Hydrazines (2a‐d) or Heterocyclic Amines (7, 12, 14, and 16).

A mixture of compound (**1**) (1 mmol) and hydrazines (**2a‐d**) or heterocyclic amines **(7, 12, 14,** and **16) (**1 mmol for each) was dissolved in absolute ethanol. The reaction mixture was refluxed at 80  °C for 4 h, with progress monitored by TLC until the starting materials were fully consumed. Upon completion, the solution was cooled, and the resulting solid product was filtered off and recrystallized from either ethanol or a mixture of ethanol and DMF to afford the pure compounds **6a–d, or 11, 13, 15,** and **17**, respectively.

### 5.3 5‐(2‐Benzylidenehydrazinyl)‐3‐(4‐Methoxyphenyl)‐1*H*‐Pyrazolo[3,4‐*d*]thiazole (6a)

Yellowish‐white powder, (0.31 g, 88%), mp = 223–225 °C; IR (KBr) *υ* = 3330 (NH), 3032 (CH=), 1603 (C = N) cm^−1^; ^1^H NMR (DMSO‐*d*_6_) *δ*: 3.92 (s, 3H, OCH_3_), 7.37–7.91 (m, 9H, Ar‐H), 8.40 (s, 1H, CH=N), 11.85 (s, 1H, NH), 12.20 (s, 1H, NH) ppm; ^13^C‐NMR (DMSO‐*d*_6_): *δ:* 52.9 (CH_3_‐O), 104.8, 115.4, 124.5, 125.2, 128.9, 129.1, 131.2, 134.8, 143.8, 148.6, 150.1, 158.2, 169.4 (Ar‐Cs, fused‐Cs and CH=N) ppm; MS, *m/z* (%) 349 (M^+^, 40), 77 (100). Anal. Calcd. For C_18_H_15_N_5_OS (349.10): C, 61.87; H, 4.33; N, 20.04; S, 9.18. Found: C, 61.69; H, 4.14; N, 20.11; S, 9.04%.

### 5.4 5‐(2‐Benzylidenehydrazinyl)‐3‐(4‐Methoxyphenyl)‐1‐Phenyl‐1*H*‐Pyrazolo[3,4‐*d*]thiazole (6b)

Creamy powder, (0.37 g, 86%), mp = 198–200 °C; IR (KBr) *υ* = 3328 (NH), 3028 (CH=), 1601 (C=N) cm^−1^; ^1^H NMR (DMSO‐*d*_6_) *δ*: 3.87 (s, 3H, OCH_3_), 7.42–7.73 (m, 14H, Ar‐H), 8.38 (s, 1H, CH=N), 11.96 (s, 1H, NH) ppm; ^13^C‐NMR (DMSO‐*d*_6_): *δ:* 53.4 (CH_3_‐O), 107.1, 114.4, 124.5, 125.2, 126.3, 128.2, 128.9, 129.1, 129.7, 131.2, 134.5, 138.4, 141.8, 143.6, 145.1, 159.2, 169.6 (Ar‐Cs, fused‐Cs and CH=N) ppm; MS, *m/z* (%) 425 (M^+^, 65), 77 (100). Anal. Calcd. For C_24_H_19_N_5_OS (425.13): C, 67.75; H, 4.50; N, 16.46; S, 7.53. Found: C, 67.89; H, 4.64; N, 16.31; S, 7.34%.

### 5.5 5‐(2‐Benzylidenehydrazinyl)‐3‐(4‐Methoxyphenyl)‐1‐(2,4‐Dinitrophenyl)‐1*H*‐Pyrazolo[3,4‐*d*] Thiazole (6c)

Brown powder, (0.43 g, 84%), mp = 247–249 °C; IR (KBr) *υ* = 3328 (NH), 3026 (CH=), 1599 (C=N) cm^−1^; ^1^H NMR (DMSO‐*d*_6_) *δ*: 3.86 (s, 3H, OCH_3_), 7.42–8.78 (m, 12H, Ar‐H), 8.36 (s, 1H, CH=N), 11.91 (s, 1H, NH) ppm; ^13^C‐NMR (DMSO‐*d*_6_): *δ:* 53.6 (CH_3_‐O), 106.9, 115.1, 120.4, 124.5, 125.2, 127.3, 128.2, 128.8, 129.5, 131.2, 134.1, 137.4, 141.6, 142.3, 143.6, 144.1, 146.7, 159.2, 169.6 (Ar‐Cs, fused‐Cs and CH=N) ppm; MS, *m/z* (%) 515 (M^+^, 25), 77 (100). Anal. Calcd. For C_24_H_17_N_7_O_5_S (515.10): C, 55.92; H, 3.32; N, 19.02; S, 6.22. Found: C, 55.83; H, 3.44; N, 18.91; S, 6.14%.

### 5.6 5‐(2‐Benzylidenehydrazinyl)‐1‐(5,5‐Diphenyl‐3,5‐Dihydro‐4‐Oxo‐Imidazol‐2‐Yl)‐3‐(4‐Methoxy Phenyl)‐ 1*H*‐Pyrazolo[3,4‐*d*]thiazole (6d)

Yellow powder, (0.47 g, 80%), mp = 213–215 °C; IR (KBr) *υ* = 3334 (NH), 3028 (CH=), 1678 (C=O), 1602 (C=N) cm^−1^; ^1^H NMR (DMSO‐*d*_6_) *δ*: 3.87 (s, 3H, OCH_3_), 7.32–7.73 (m, 19H, Ar‐H), 8.38 (s, 1H, CH=N), 9.98 (s, 1H, NH), 11.66 (s, 1H, NH) ppm; ^13^C‐NMR (DMSO‐*d*_6_): *δ:* 53.9 (CH_3_‐O), 82.8 (C(Ph)_2_), 110.1, 114.9, 125.2, 126.3 (2C), 127.4 (2C), 128.2 (2C), 129.4 (3C), 131.2, 133.5, 138.4, 141.8 (2C), 143.6, 145.1, 157.2, 160.1, 169.6 (Ar‐Cs, fused‐Cs and CH=N), 176.4 (C=O) ppm; MS, *m/z* (%) 583 (M^+^, 40), 77 (100). Anal. Calcd. For C_33_H_25_N_7_O_2_S (583.18): C, 67.91; H, 4.32; N, 16.80; S, 5.49. Found: C, 67.77; H, 4.24; N, 16.71; S, 5.38%.

### 5.7 7‐(2‐Benzylidenehydrazinyl)‐5‐(4‐Methoxyphenyl)thiazolo[5,4‐*e* [1,2,4] Triazolo[1,5‐*a*]pyrimidine (11)

Creamy powder, (0.33 g, 82%), mp = 2331–233 °C; IR (KBr) *υ* = 3328 (NH), 3028 (CH=), 1601 (C=N) cm^−1^; ^1^H NMR (DMSO‐*d*_6_) *δ*: 3.86 (s, 3H, OCH_3_), 7.08–7.72 (m, 9H, Ar‐H), 8.37 (s, 2H, CH=N and CH‐triazole), 11.97 (s, 1H, NH) ppm; ^13^C‐NMR (DMSO‐*d*_6_): *δ:* 53.8 (CH_3_‐O), 109.1, 114.4, 125.5, 126.3, 128.2, 129.5, 131.2, 134.1, 143.6, 154.1, 156.3, 158.2, 160.4, 161.8. 169.6 (Ar‐Cs, fused‐Cs and CH=N) ppm; MS, *m/z* (%) 401 (M^+^, 60), 77 (100). Anal. Calcd. For C_20_H_15_N_7_OS (401.11): C, 59.84; H, 3.77; N, 24.42; S, 7.99. Found: C, 59.98; H, 3.64; N, 24.31; S, 8.04%.

### 5.8 2‐(2‐Benzylidenehydrazinyl)‐4‐(4‐Methoxyphenyl)benzoimidazo[1,2‐*a*]thiazolo[5,4‐*e*]pyrimidine (13)

Yellow powder, (0.36 g, 80%), mp = 202–204 °C; IR (KBr) *υ* = 3334 (NH), 3065 (CH=), 1603 (C=N) cm^−1^; ^1^H NMR (DMSO‐*d*_6_) *δ*: 3.86 (s, 3H, OCH_3_), 7.05–7.74 (m, 13H, Ar‐H), 8.37 (s, 1H, CH=N), 11.97 (s, 1H, NH) ppm; ^13^C‐NMR (DMSO‐*d*_6_): *δ:* 54.1 (CH_3_‐O), 108.7, 112.4, 114.4, 120.5, 122.2, 123.7, 125.5, 128.1, 128.7, 129.5, 131.2, 133.9, 135.4, 141.6, 144.1, 150.4, 158.2, 160.4, 164.8. 169.6 (Ar‐Cs, fused‐Cs and CH=N) ppm; MS, *m/z* (%) 450 (M^+^, 45), 77 (100). Anal. Calcd. For C_25_H_18_N_6_OS (450.13): C, 66.65; H, 4.03; N, 18.65; S, 7.12. Found: C, 66.88; H, 3.94; N, 18.41; S, 7.03%.

### 5.9 2‐(2‐Benzylidenehydrazinyl)‐4‐(4‐Methoxyphenyl)‐4*H*‐Benzothiazolo[3,2‐*a*]thiazolo[5,4‐*e*] Pyrimidine (15)

Yellowish‐white powder, (0.37 g, 80%), mp = 211–213 °C; IR (KBr) *υ* = 3334 (NH), 3060 (CH=), 2961 (CH), 1602 (C=N) cm^−1^; ^1^H NMR (DMSO‐*d*_6_) *δ*: 3.86 (s, 3H, OCH_3_), 7.08–7.92 (m, 13H, Ar‐H), 5.18 (s, 1H, CH‐Ar), 8.36 (s, 1H, CH=N), 11.96 (s, 1H, NH) ppm; ^13^C‐NMR (DMSO‐*d*_6_): *δ:* 54.2 (CH_3_‐O), 67.4 (CH‐Ar), 114.7, 117.4, 118.4, 122.2, 122.8, 126.7, 127.5, 128.1, 129.4, 131.2, 132.8, 133.4, 134.6, 137.1, 139.4, 143.2, 158.4, 159.8, 169.4 (Ar‐Cs, fused‐Cs and CH=N) ppm; MS, *m/z* (%) 469 (M^+^, 40), 77 (100). Anal. Calcd. For C_25_H_19_N_5_OS_2_ (469.10): C, 63.95; H, 4.08; N, 14.91; S, 13.65. Found: C, 63.88; H, 3.95; N, 14.71; S, 13.42%.

### 5.10 2‐(2‐Benzylidenehydrazinyl)‐4‐(4‐Methoxyphenyl)‐4*H*‐Pyrido[1,2‐*a*]thiazolo[5,4‐*e*]pyrimidine(17)

Yellow powder, (0.33 g, 80%), mp = 186–188 °C; IR (KBr) *υ* = 3331 (NH), 3063 (CH=), 2958 (CH), 1601 (C=N) cm^−1^; ^1^H NMR (DMSO‐*d*_6_) *δ*: 3.85 (s, 3H, OCH_3_), 7.04–8.12 (m, 13H, Ar‐H), 5.19 (s, 1H, CH‐Ar), 8.37 (s, 1H, CH=N), 11.95 (s, 1H, NH) ppm; ^13^C‐NMR (DMSO‐*d*_6_): *δ:* 54.7 (CH_3_‐O), 66.4 (CH‐Ar), 104.5, 114.7, 122.3, 127.5, 128.4, 129.4, 131.2, 132.6, 133.4, 134.3, 135.7, 137.1, 143.2, 147.5, 158.4, 159.8, 169.4 (Ar‐Cs, fused‐Cs and CH=N) ppm; MS, *m/z* (%) 413 (M^+^, 25), 77 (100). Anal. Calcd. For C_23_H_19_N_5_OS (314.13): C, 66.81; H, 4.63; N, 16.94; S, 7.75. Found: C, 66.68; H, 4.55; N, 16.78; S, 7.62%.

### 5.11 Frontier Molecular Orbitals and Overall Reactivity Descriptors

The frontier molecular orbitals and global reactivity descriptors of the studied compounds were obtained at the same computational level. Widely recognized qualitative chemical concepts from conceptual DFT were employed to assess both the reactivity and stability of the system.^[^ ^64^ ^,^ ^65^ ^]^

### 5.12 In Silico Studies: Drug Likeness and ADMET Prediction

The online SwissADME service was utilized to evaluate the pharmacokinetic properties and drug‐like properties of the synthesized compounds.^[^ ^76^ ^,^ ^77^ ^]^ The toxicity profile for all synthesized compounds was evaluated using pkCSM. (https://biosig.lab.uq.edu.au/pkcsm/ prediction).^[^ ^72^ ^,^ ^78^ ^]^

### 5.13 Molecular Docking Studies of Thiazole Derivatives

The three‐dimensional molecular structures of the most active compounds, along with their standard bond lengths and angles, were built using ChemBio3D Ultra 14.0 (Cambridge Soft Corporation, USA). The crystal structure of Staphylococcus aureus proteins was obtained from the Protein Data Bank (PDB) for molecular docking studies. Co‐crystallized ligands and water molecules were removed, while polar hydrogens and Kollman‐united charges were assigned to the receptor. Ligand and protein database files were prepared using MOE software.^[^ ^79^ ^,^ ^80^ ^]^

## 6 Conclusion

We have developed a concise and regioselective aza‐Michael cyclization protocol for the synthesis of fused pyrazolo[3,4‐d]thiazoles and related polycyclic thiazolo heterocycles. A single electrophilic enone substrate bearing two Michael acceptor sites undergoes selective 1,4‐addition with diverse hydrazines and heterocyclic amines under mild reflux conditions to furnish exclusively the 1H‐regio‐isomers in good to excellent yields. DFT analyses of frontier molecular orbitals, parr local reactivity indices, and MEP maps predicted the higher electrophilicity of the olefinic C=C bond and the nucleophilicity of amine nitrogen centers, explaining the observed regioselectivity. Global Reactivity Descriptors confirmed that the kinetically and thermodynamically favored products align with our proposed mechanistic pathway. Molecular docking against key Gram‐positive (2IWC), Gram‐negative (2NXW), and anticancer (4OH4) protein targets demonstrated that several synthesized compounds bind favorably—often engaging the same active‐site residues as standard drugs—and exhibit binding energies comparable to or better than those references. In silico ADME/T profiling revealed compliance with Lipinski's rules, high predicted oral bioavailability, negligible blood–brain barrier permeability, acceptable toxicity profiles, and synthetic accessibility scores comparable to marketed drugs, supporting the feasibility of large‐scale synthesis. These findings highlight the promise of these heterocycles as lead scaffolds for further antibacterial and anticancer evaluation.

## Conflict of Interest

The authors declare no conflict of interest.

## Data Availability

The data that support the findings of this study are available from the corresponding author upon reasonable request.
